# Tryptophan in Nutrition and Health 2.0

**DOI:** 10.3390/ijms24087112

**Published:** 2023-04-12

**Authors:** Burkhard Poeggeler, Sandeep Kumar Singh, Kumar Sambamurti, Miguel Angelo Pappolla

**Affiliations:** 1 Department of Physiology, Johann-Friedrich-Blumenbach-Institute for Zoology and Anthropology, Faculty of Biology Georg August University Göttingen, Göttingen and Goettingen Research Campus, Am Türmchen 3, D-33332 Gütersloh, Germany; 2 Indian Scientific Education and Technology Foundation, Lucknow 226002, India; sandeeps.bhu@gmail.com; 3 Department of Neurobiology, Medical University of South Carolina, 173 Ashley Avenue, BSB 403, Charleston, SC 29425, USA; sambak@musc.edu; 4 Department of Neurology, University of Texas Medical Branch, 301 University Boulevard, Galveston, TX 77555, USA; pappolla@aol.com

This editorial summarizes the eight articles that have been collected for the Special Issue entitled “Tryptophan in Nutrition and Health 2.0,” and demonstrates their relevance to the field. Tryptophan is a rate-limiting essential amino acid, the metabolites of which are important endogenous molecular mediators of physiology and pathophysiology (Figure 1).

Tryptophan deficiency manifests itself rapidly under stress, inflammation, and catabolic conditions [1]. Modulation of the tryptophan metabolism can prevent age-related diseases, including cognitive and physical decline [1,2]. Endogenous metabolites, such as indole-3-propionic acid, can act as potent protective agents [2]. The kynurenine-to-tryptophan ratio constitutes a novel relevant biomarker for assessing organism and ecosystem health [3]. UVB enhances the antiproliferative activity of kynurenine and kynurenic acid in melanoma cells [4]. The measurement of altered metabolites in melanoma patients can be used to improve diagnosis and treatment assessment [5]. The neuroprotective tryptophan derivative melatonin can determine the brain–heart crosstalk [6]. New data suggest a modulatory role of serotonin biosynthesis in the reprogramming of somatic cells to a pluripotent state [7]. Activation of the kynurenine and indolamine pathways of the tryptophan metabolism is linked to a plethora of neuropsychiatric disorders [8]. Transcranial magnetic stimulation can elevate brain serotonin levels, thereby restoring normal neurotransmission. The tryptophan metabolism can be influenced to prevent and reverse premature aging characterized by inflammation and oxidative stress [1,2]. Targeting tryptophan and the tryptophan pathway can enable novel strategies for diagnosis, prevention, treatment, and rehabilitation to improve, maintain, and restore health [1,2,3,4,5,6,7,8].

## Figures and Tables

**Figure 1:**
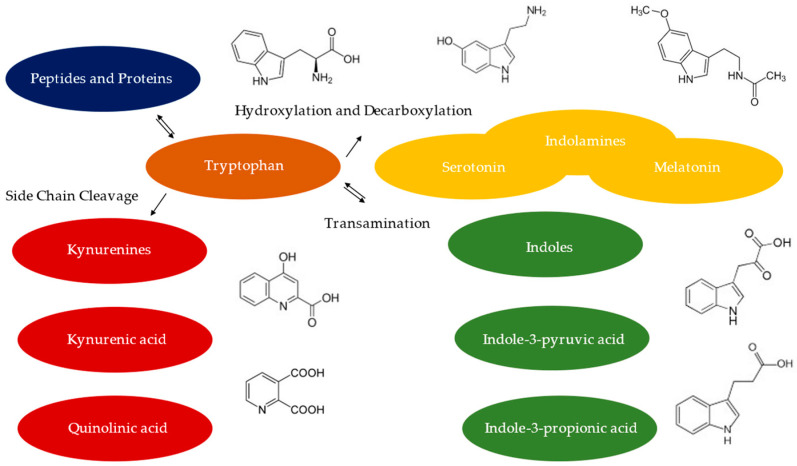
Three tryptophan pathways leading to endogenous indolamines, kynurenines and indoles.

## Data Availability

Not applicable.
